# Strong associations and moderate predictive value of early symptoms for SARS-CoV-2 test positivity among healthcare workers, the Netherlands, March 2020

**DOI:** 10.2807/1560-7917.ES.2020.25.16.2000508

**Published:** 2020-04-23

**Authors:** Alma Tostmann, John Bradley, Teun Bousema, Wing-Kee Yiek, Minke Holwerda, Chantal Bleeker-Rovers, Jaap ten Oever, Corianne Meijer, Janette Rahamat-Langendoen, Joost Hopman, Nannet van der Geest-Blankert, Heiman Wertheim

**Affiliations:** 1 Department of Medical Microbiology, Radboud Centre for Infectious Diseases, Radboud university medical centre, Nijmegen, The Netherlands; 2 MRC Tropical Epidemiology Group, London School of Hygiene and Tropical Medicine, London, United Kingdom; 3 Department of Infection and Immunity, London School of Hygiene and Tropical Medicine, London, United Kingdom; 4 Department of Internal Medicine, Radboud Centre for Infectious Diseases, Radboud university medical centre, Nijmegen, The Netherlands; 5 Department of Occupational Health, Radboud university medical centre, Nijmegen, The Netherlands

**Keywords:** outbreak, SARS-CoV-2, COVID-19, pandemic, screening, healthcare workers, prediction model

## Abstract

Healthcare workers (n = 803) with mild symptoms were tested for severe acute respiratory syndrome coronavirus 2 (SARS-CoV-2) (n = 90 positive) and asked to complete a symptom questionnaire. Anosmia, muscle ache, ocular pain, general malaise, headache, extreme tiredness and fever were associated with positivity. A predictive model based on these symptoms showed moderate discriminative value (sensitivity: 91.2%; specificity: 55.6%). While our models would not justify presumptive SARS-CoV-2 diagnosis without molecular confirmation, it can contribute to targeted screening strategies.

Following the emergence of severe acute respiratory syndrome coronavirus 2 (SARS-CoV-2) in China in December 2019, countries worldwide strive to contain or slow down virus transmission to allow health facilities to cope with the rapid rise of patients with coronavirus disease (COVID-19) [1,2]. In February 2020, the first patient with COVID-19 was reported in the Netherlands. Cases initially clustered in the province of Noord Brabant, followed by dissemination across the country [3].

Healthcare workers (HCW) are disproportionally at risk of contracting SARS-CoV-2 [4]. To protect HCW and prevent nosocomial transmission, it is recommended that healthcare facilities have a strategy for testing, management and follow-up of HCW with respiratory symptoms [2,4]. Test results also guide when an HCW can return to work with mild symptoms. Unfortunately, national and institutional strategies are in part dependent on the operational feasibility.

Here, we present our findings from a large cohort of symptomatic HCW who were tested for SARS-CoV-2 infection as part of our hospital programme. We aimed to identify symptoms associated with test positivity and develop a diagnostic model to predict SARS-CoV-2 infection based on early symptoms. In a context of limited availability of testing supplies, prediction models may support targeted testing strategies and guidelines to refrain from or resume clinical duties.

## Screening of healthcare workers

Since early March 2020, HCW in our hospital have been tested for SARS-CoV-2 when they have symptoms suggestive of COVID-19 according to institutional policy based on the latest evidence. All 1,247 HCW tested between 10 and 29 March 2020 received an email with a link to an online anonymous questionnaire on the symptoms they experienced before their test. We received 803 completed questionnaires, an overall response rate of 64%. By 29 March, there had been 112 HCW with a positive test, of whom 90 responded to the questionnaire, which suggests a slight overrepresentation of test-positives among the respondents (response rate 80%).

Initially, the questionnaire covered respiratory and general non-respiratory symptoms and was completed by 627 HCW (56 SARS-CoV-2-positive) between 10 and 23 March 2020. After reports on anosmia and gastrointestinal illness in the initial COVID-19 patients, the questionnaire was adapted on 24 March to also include anosmia, diarrhoea, nausea and extreme tiredness. This updated questionnaire was completed by 176 HCW (34 positives) between 24 and 30 March 2020. Most respondents were female (82.9%) and between 21 and 40 years-old (55.7%) (Table 1). 

**Table 1:** Description of the study population of healthcare workers tested for SARS-CoV-2, by PCR result, the Netherlands, March 2020 (n = 803)

	Total n = 803		SARS-CoV-2-positive n = 90		SARS-CoV-2-negative n = 713	
	n	%	n	%	n	%
**Sex^a^**						
Male	141	17.6	19	21.1	122	17.1
Female	661	82.4	71	78.9	590	82.9
**Age group (years)**						
< 20	10	1.2	0	0.0	10	1.4
21–30	216	26.9	24	26.7	192	26.9
31–40	231	28.8	22	24.4	209	29.3
41–50	173	21.5	25	27.8	148	20.8
51–60	133	16.6	16	17.8	117	16.4
> 60	40	5.0	3	3.3	37	5.2
**Comorbidities (one or more)**						
Chronic lung disease	61	7.6	4	4.4	57	8.0
Disease of immune system	29	3.6	0	0.0	29	4.1
Allergy	137	17.1	8	8.9	129	18.1
**Medical profession**						
Medical doctor	144	17.9	21	23.3	123	17.3
Nurse	266	33.1	31	34.4	235	33.0
Other healthcare worker	393	48.9	38	42.2	355	49.8
**Questionnaire**						
Initial questionnaire (for development of model)	627	78.1	56	62.2	571	80.1
Extended questionnaire (for validation of the model)	176	21.9	34	37.8	142	19.9

SARS-CoV-2: severe acute respiratory syndrome coronavirus 2.

**^a^** For one person information on sex was missing.

To allow a diagnostic model to be created based on the initial cohort (627 HCW) and tested in the second cohort (176 HCW), all HCW who were tested positive before 24 March (n = 77) and a random selection (n = 99) of the HCW who tested negative before 24 March received an additional questionnaire asking about these four additional symptoms separately. This additional questionnaire was completed by 45 SARS-CoV-2-positive and 48 negative HCW.

## Symptoms associated with SARS-CoV-2-positive test results

The most frequently reported symptoms among test-negative HCW were cough (60%), sore throat (56%) and common cold (51%). Test-positive HCW most frequently reported headache (71%), general malaise (63%) and muscle ache (63%) (Table 2). Among 176 HCW who responded to the second questionnaire after 24 March, HCW who tested positive for SARS-CoV-2 had a median of four symptoms (interquartile range (IQR): 2–6) compared with three symptoms (IQR: 2–5) for HCW who tested negative (p = 0.004).

**Table 2:** Univariate associations of early symptoms with SARS-CoV2 PCR positivity among healthcare workers, the Netherlands, March 2020 (n = 803)

Symptom	SARS-CoV2-positive		SARS-CoV2-negative		OR (95% CI)	p value
	n/N	%	n/N	%		
**General non-respiratory symptoms**						
Anosmia^a^	37/79	46.8	7/190	3.7	23.0 (8.2­–64.8)	< 0.001
Muscle ache	57/90	63.3	143/713	20.1	6.9 (4.2–11.3)	< 0.001
Ocular pain	31/90	34.4	75/713	10.5	4.5 (2.7–7.4)	< 0.001
General malaise	57/90	63.3	208/713	29.2	4.2 (2.6–6.7)	< 0.001
Headache	64/90	71.1	296/713	41.5	3.5 (2.1–5.7)	< 0.001
Extreme tiredness^a^	45/79	57.0	61/190	32.1	2.8 (1.6–4.9)	< 0.001
Fever	51/90	56.7	233/713	32.7	2.7 (1.7–4.2)	< 0.001
**Respiratory symptoms**						
Common cold	50/90	55.6	363/713	50.9	1.2 (0.8–1.9)	0.406
Sneeze	36/90	40.0	253/713	35.5	1.2 (0.8–1.9)	0.401
Cough	53/90	58.9	424/713	59.5	1.0 (0.6–1.5)	0.916
Shortness of breath	20/90	22.2	157/713	22.0	1.0 (0.6–1.7)	0.965
Runny nose	24/90	26.7	231/713	32.4	0.8 (0.5–1.2)	0.271
Sore throat	36/90	40.0	400/713	56.1	0.5 (0.3–0.8)	0.004
**Gastrointestinal symptoms**						
Nausea^a^	13/79	16.5	17/190	8.9	2.0 (0.9–4.4)	0.075
Diarrhoea^a^	14/79	17.7	20/190	10.5	1.8 (0.9–3.9)	0.106

SARS-CoV-2: severe acute respiratory syndrome coronavirus 2.

^a^ The total here includes only those respondents of the second response cohort (34 cases and 142 non-cases) and those from the first cohort who responded to the additional questions (45 cases and 48 non-cases).

**Figure 1:**
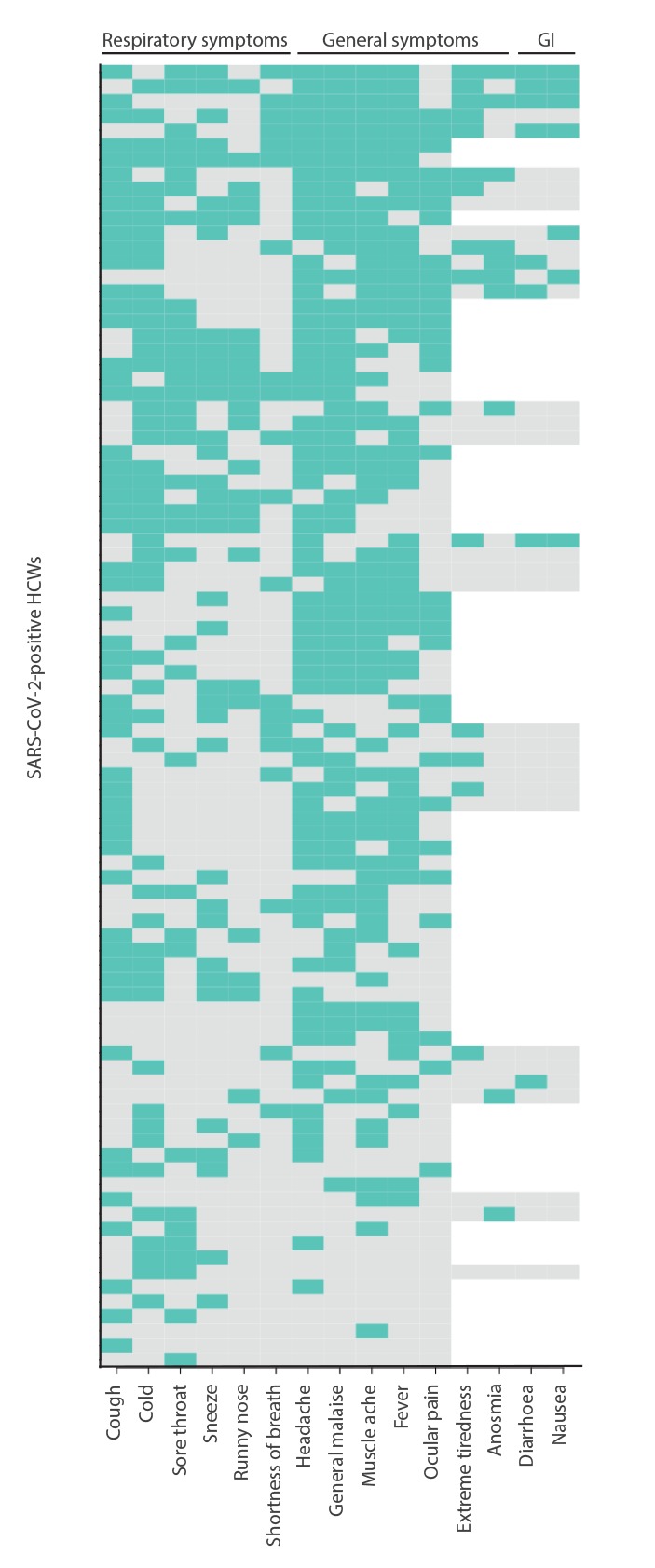
Heatmap of early symptoms reported by healthcare workers positive for SARS-CoV-2, the Netherlands, March 2020 (n = 90)

GI: gastrointestinal; SARS-CoV-2: severe acute respiratory syndrome coronavirus 2.

Univariate associations were assessed by calculating odds ratios. General non-respiratory symptoms (muscle ache, ocular pain, general malaise, headache and extreme tiredness) were associated with test positivity. Anosmia was reported by 47% of test-positives and was strongly associated with SARS-CoV-2 positivity (OR = 23.0; 95% confidence interval (CI): 8.2–64.8). Among the 14 people with anosmia in whom other symptoms were also recorded, 10 reported a runny nose and/or sneezing, while four did not report any symptoms that can cause nasal congestion. Among 10 individuals with both anosmia and muscle ache, nine were SARS-CoV-2-positive. None of the respiratory symptoms were associated with SARS-CoV-2 positivity, sore throat being less common among test positives (40.0% vs 56.1%; p = 0.004). Symptoms reported by test-positive HCW are presented in a heat map indicating which symptoms were reported together (Figure 1). 

Symptoms were grouped as respiratory, general and gastrointestinal symptoms. Green: symptom is present; grey: symptom is absent; blank: not recorded.

**Figure 2:**
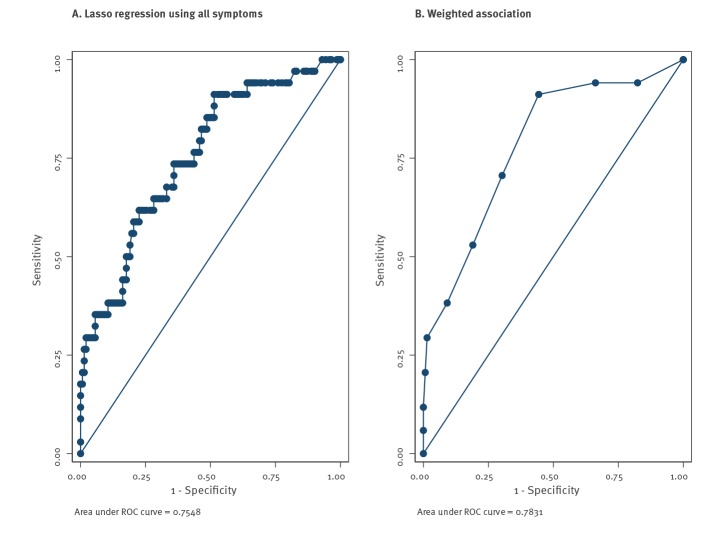
ROC curves predicting SARS-CoV-2 test results of healthcare workers (cohort 2; n = 176) based on reported symptoms in cohort 1 (n = 627), the Netherlands, March 2020

Data from the initial cohort (627 HCW) were used to predict the SARS-CoV-2 test result in the second cohort (176 HCW). A prediction model was fit on all symptoms using Lasso regression (STATA version 16.0; Statacorp, Texas, United States) [5]. This is a technique that attempts to reduce overfitting by shrinking coefficients. Some coefficients are shrunk to zero and hence effectively removed from the model. The model retained all variables except fever and cough. This model achieved an area under the ROC (receiver operating characteristic) curve of 0.754 (95% CI: 0.662–0.846) (Figure 2A) and achieved a sensitivity of 82.4% and specificity of 59.2%. A simple model which included all the symptoms with a significant positive association, weighted based on the results in Table 2 (weight of 3 for anosmia, 2 for muscle ache and 1 each for extreme tiredness, headache, ocular pain fever, and general malaise) achieved an area under the ROC curve of 0.783 (95% CI: 0.696–0.870) (Figure 2B). Using a cut-off of ≥ 3 symptoms gave a sensitivity of 91.2% and a specificity of 55.6% for SARS-CoV-2 test positivity.

ROC curve: receiver operating characteristic curve; SARS-CoV-2: severe acute respiratory syndrome coronavirus 2.

Weighting applied in panel B: 3 for anosmia, 2 for muscle ache and 1 each for extreme tiredness, headache, ocular pain, fever and general malaise.

## Discussion

This study provides valuable insights in the early symptoms of COVID-19 in a large cohort of HCW. General non-respiratory symptoms (muscle ache, ocular pain, general malaise, headache, extreme tiredness and fever) were most frequently reported by test-positive HCW and these symptoms were strongly associated with SARS-CoV-2 test positivity, unlike respiratory symptoms such as cough and sneezing. Anosmia was particularly strongly associated with test positivity. Fever and cough have been reported as early symptoms in mild COVID-19 cases [6], and fever occurs in the majority of individuals hospitalised for COVID-19 [7]. However, in our study fever was not the strongest predictor of test positivity among HCW, which is in line with findings from a similar study from the Netherlands [8].

Recent work from the Netherlands showed that 63% of HCW had worked despite mild symptoms [8]. Clear guidance on the use of personal protective equipment (PPE) and clear protocols for work abstinence during symptoms are vital to protect HCW and patients. Models of triage and diagnosis may help physicians and public health authorities to estimate infection risk and support decisions about such isolation and quarantine measures [9]. Our simple prediction model based on the seven symptoms most strongly associated with SARS-CoV-2 positivity among HCW, giving extra weight to anosmia and muscle ache as strongest predictors, gave a high sensitivity (91.2%) and a moderate specificity (55.6%). Where the sensitivity and specificity of a diagnostic test or prediction algorithm are features of the test, the positive predictive value (PPV) and negative predictive value (NPV) are related to the infection prevalence in the population. With increasing prevalence, the PPV increases and NPV decreases. With a prevalence of infection of 10%, the above sensitivity and specificity would result in a PPV of 16.9% and NPV of 98.5%. This is very informative, as with only 1.5% chance of having SARS-CoV-2 upon a negative ‘prediction’, hospitals could consider letting those HCW work (with PPE), depending on the shortage of staff. Because of the low specificity, about half of HCW would be predicted as ‘positive’. However, testing only this subgroup of ‘predicted positives’ would reduce the necessary testing materials by 50%.

A unique aspect of our study is that we assessed early symptoms of COVID-19 in an otherwise healthy population. The HCW received the invitation to the questionnaire the day after receiving their test result, which was usually within 24 h after the sample was taken, thus minimising recall bias. The response rate was 64%, and selection bias could have occurred as people with atypical (i.e. non-respiratory symptoms) could have been more likely to respond to the questionnaire, leading to an overrepresentation of people with these symptoms. However, as the percentage of respiratory symptoms among test-positive and test-negative HCW was comparable, we do not expect that this could have explained the strong associations observed in this study. Our anonymous data collection did not allow us to examine initial symptoms in relation to disease progression. The study design would have allowed for the calculation of relative risks (RR), were it not that we had asked for four symptoms in a case–control manner. Even though RR are easier to interpret than OR, and OR may slightly overestimate the risk estimate, presenting a mix of RR and OR would have been confusing. In addition, presenting the OR with column percentages gives the useful insight of the percentage of cases (and non-cases) that reported a certain symptoms.

## Conclusion

Our findings may be used to refine national public health guidelines on self-isolating individuals whose symptoms are suggestive of COVID-19. The current policy in the Netherlands is that people with mild respiratory symptoms self-isolate and household members of people with fever are requested to self-quarantine. Based on our findings, this could be expanded with general non-respiratory symptoms or anosmia. Our detailed report of early symptoms among HCW tested for SARS-CoV-2 identified that general non-respiratory symptoms and anosmia were strongly associated with test positivity. While our prediction models would not justify presumptive SARS-CoV-2 diagnosis without molecular confirmation, findings may contribute to a targeted screening strategy which may be of value in settings with limited availability of testing materials.
